# Childhood trauma and genetic variation in the *DAT* 40-bp VNTR contribute to HIV-associated neurocognitive disorders

**DOI:** 10.1016/j.ibneur.2021.12.003

**Published:** 2021-12-08

**Authors:** Aqeedah Abbas Roomaney, Jacqueline Samantha Womersley, Patricia Cathryn Swart, Georgina Spies, Soraya Seedat, Sian Megan Joanna Hemmings

**Affiliations:** a Division of Molecular and Human Genetics, Department of Biomedical Sciences, Faculty of Medicine and Health Sciences, Stellenbosch University, Cape Town, South Africa; b Department of Psychiatry, Faculty of Medicine and Health Sciences, Stellenbosch University, Cape Town, South Africa; c South African Medical Research Council / Stellenbosch University Genomics of Brain Disorders Research Unit, Faculty of Medicine & Health Sciences, Stellenbosch University, Cape Town, South Africa; d South African Research Chair in PTSD, Department of Psychiatry, Faculty of Medicine & Health Sciences, Stellenbosch University, Cape Town, South Africa

**Keywords:** HIV, Human immunodeficiency virus, COMT, Catechol-O-methyltransferase, DAT, Dopamine transporter, HAND, HIV-associated neurocognitive disorders, HIV, HIV-associated neurocognitive disorders, Dopamine transporter, Catechol-O-methyltransferase, Childhood trauma

## Abstract

HIV/AIDS is a major public health burden in South Africa, currently affecting an estimated 13.5% of the population. Despite improved access to antiretroviral therapies, HIV-associated neurocognitive disorders (HAND), characterised by a spectrum of neurocognitive impairment, emotional disturbances and motor abnormalities, continue to persist. Gene-environment interactions contribute to HAND pathophysiology and previous research has identified childhood trauma as an environmental risk factor. Dopaminergic signalling in the prefrontal cortex plays a key role in cognitive function. Thus, variants in genes encoding the dopamine transporter (DAT) and catechol-O-methyltransferase (COMT), which are responsible for dopamine transport and metabolism, could represent genetic risk factors for HAND. This study investigated whether the *DAT* variable number of tandem repeats (VNTR) and *COMT* Val158Met (rs4680) polymorphisms are associated with longitudinal change in cognitive function in the context of childhood trauma and HIV. Participants (*n* = 49 HIV-negative and *n* = 64 HIV-positive women) completed the Childhood Trauma Questionnaire – Short Form (CTQ-SF) and provided blood for genetic analyses. Global cognitive scores were generated from baseline and one-year follow-up assessments. Following polymerase chain reaction, genotypes were determined using gel electrophoresis and confirmed by Sanger sequencing. Baseline global cognitive scores, genotype, HIV status and CTQ-SF scores were regressed on one-year global cognitive scores in regression models. Analysis of variance was used to examine the effect of including predictor variable interactions on model fit. HIV seropositivity was associated with poorer cognitive performance at one-year follow-up (*p* = 2.46 ×10^−4^). The combination of HIV and *DAT* 10-repeat homozygosity (*DAT* 10/10) was associated with reduced global cognitive scores in longitudinal models (*p* = 0.010). Including the interaction between *DAT* 10/10, childhood trauma, and HIV explained significantly more of the variance in longitudinal cognitive scores (*p* = 0.008). There were no significant associations with the *COMT* genotype. Our research indicates that childhood trauma and genetic variation in *DAT* contribute toward the aetiology of HAND. Future studies in larger cohorts are warranted to verify these results.

## Highlights

• Gene-environment interactions influence cognitive function in HIV.
• The interaction of childhood trauma with DAT genetic variation contributes to HAND.
• HIV x DAT 10/10 was associated with reduced longitudinal cognitive scores.

## 1 Introduction

### 1.1 HIV-associated neurocognitive disorders

Though neurocognitive consequences of HIV infection are common, the exact burden of HIV-associated neurocognitive disorders (HAND) is challenging to determine. Prevalence estimates range from 20% to 69%, with factors such as assessment tools, study location and antiretroviral therapy use likely influencing these rates (Nightingale et al., 2014). A meta-analysis involving 13,513 participants from 32 countries described an overall prevalence of HAND of 42.6%, with sub-Saharan Africa accounting for an estimated 72% of cases (Wang et al., 2020). In 2019, HIV was estimated to affect 19% of South African adults with a prevalence of 25% in South African women, compared to 12.9% in South African men of the same age (UNAIDS, 2019). The high rate of intimate partner violence, rape and childhood trauma contribute to the high prevalence of HIV amongst South African women (Andersson et al., 2008, Jewkes, 2002, Jewkes et al., 2001, Kalichman and Simbayi, 2004). HIV infection puts individuals at particularly high risk for developing HAND.

HAND involves cognitive impairment, emotional disturbances and motor abnormalities that occur as a result of HIV-induced neurodegeneration and neurotransmitter dysregulation (Abassi et al., 2017). HAND may be asymptomatic or symptomatic. Symptomatic HAND may present as mild neurocognitive disorder or HIV-associated dementia (HAD) (Antinori et al., 2007), characterised by decreased attention, psychomotor slowing, motor dysfunction and working memory deficits (Antinori, 2007, Chaganti, 2017, Heaton, 2011, Sacktor, 2018). The introduction of combined antiretroviral therapy, which suppresses HIV replication, has resulted in a significant decrease in the incidence of HAD (Maschke et al., 2000). However, less severe forms of HAND persist, affecting up to 52% of people living with HIV/AIDS (Chaganti et al., 2017). This may be partly due to the limited penetration of the blood-brain barrier by certain antiretrovirals (e.g. HIV protease inhibitors and nucleoside analogues) (Cunningham et al., 2000, Yuan and Kaul, 2019). Evidence for the effectiveness of blood-brain barrier penetration of antiretrovirals on cognition is equivocal, with both higher and lower central nervous system (CNS) penetration effectiveness of antiretrovirals associated with cognitive improvements, whilst some studies have found no association between CNS penetration effectiveness and cognitive benefits (Yuan and Kaul, 2019).

HIV-induced CNS damage may contribute to the neurocognitive decline experienced by individuals with HAND (Antinori et al., 2007). Neuropathological effects are induced when HIV infected monocytes and T cells infect brain cells after their migration across the blood brain barrier into the CNS (Atluri et al., 2015). These cells produce proinflammatory cytokines, such as tumor necrosis factor (TNF) and interleukin-1 beta (IL-1ꞵ), which further activate microglia and astrocytes, leading to neuroinflammation and the release of neurotoxic excitatory amino acids and inflammatory mediators. This cascade eventually results in neuronal dysfunction and cell death, and is associated with a characteristic set of cognitive, emotional, and motor impairments experienced by patients with HAND (Everall, 1999, Hong and Banks, 2015).

The diagnosis of HAND involves several neurocognitive tests, which assess functioning on several cognitive domains, namely verbal or language, attention / working memory, executive functioning, memory (learning and recall), speed of information processing, sensory perception, and motor skill domains, from which a global neurocognitive score is calculated (Antinori et al., 2007). These tests are standardised across linguistic, cultural and educational backgrounds and scores are then compared to demographically adjusted norms, as appropriate (Antinori et al., 2007). Although these tests provide a diagnosis of HAND, biomarkers that could identify preclinical HAND or predict rapid cognitive decline would be beneficial in understanding HAND in countries with a high HIV prevalence, such as South Africa. This would help identify individuals at a higher risk for developing HAND in order to provide treatment at the earliest stage of neurological decline (Saylor et al., 2016). The neuropathological features of HAND do not necessarily accord with detectable impairments on neurocognitive testing. For example, elevated viral load in the cerebrospinal fluid (CSF) may be linked with symptoms of HAND, even when plasma viral load is undetectable (Khoury et al., 2013). While CSF viral load is a better predictor of the symptoms of HAND than plasma viral load, this association is not robust. The pathophysiology of HAND therefore extends beyond viral-induced neurotoxicity.

### 1.2 Childhood trauma contributes to HAND

Environmental stressors and modifiable risk factors, such as childhood trauma, have been associated with the progression of HAND (Spies et al., 2017). Childhood trauma refers to emotional, physical, or sexual abuse or physical or emotional neglect experienced by a child (Turner et al., 2010). Changes in brain morphometry have been associated with HIV and childhood trauma, with smaller volumes observed in the right anterior cingulate cortex, bilateral hippocampi, corpus collosum, left and right caudate, and putamen of HIV infected women with childhood trauma (Spies et al., 2016). Shared pathophysiological mechanisms underlying childhood trauma and HIV may contribute toward HAND (Spies, 2017, Womersley et al., 2017). A longitudinal study examining the effects of childhood trauma and HIV on neurocognitive function suggests poorer cognitive performance in South African women dually affected with HIV and childhood trauma, with the combined effect of HIV and childhood trauma shown to be associated with reduced executive functions and verbal fluency (Spies et al., 2017).

Similar findings were seen in all-male cohorts as well, with neurocognitive impairment in males living with HIV significantly associated with trauma exposure (Deiss, 2019, Pukay-Martin, 2003). Potentially traumatic events and stressful life events were associated with HIV-related deficits in executive functioning, verbal fluency, attention / working memory, processing speed, motor function, and global neurocognitive impairment in a cohort of HIV-positive and -negative males with and without trauma exposure (Pukay-Martin et al., 2003). A lifetime history of posttraumatic stress disorder was associated with neurocognitive impairment in a cohort of 189 male United States military personnel living with HIV, with these participants six times more likely to develop HIV-related neurocognitive impairment than individuals without posttraumatic stress disorder (Deiss et al., 2019).

### 1.3 Dopamine signalling and HAND

Decreased connectivity between brain regions that form the salience network, such as the interior insula, anterior cingulate, amygdala, susbstantia nigra, and thalamus, and the executive network, which connects the dorsolateral prefrontal cortex to the striatum and parietal areas, have been observed in individuals with HAND (Chaganti et al., 2017). Changes in the connectivity of these regions correlate with the degree of neurocognitive impairment observed (Chaganti et al., 2017). HIV is associated with premature aging of the salience network, which is important in determining the salience of external stimuli and internal brain events (Chaganti, 2017, Thomas, 2013). This network is closely linked to the mesolimbic dopamine system and is therefore strongly influenced by dopaminergic function (McCutcheon et al., 2019).

Dopamine is a neurotransmitter that is associated with reward systems of the brain and plays a role in modulating learning and motivation (Berke, 2018). Dopamine binds to dopamine receptors and regulates, amongst others, emotion, cognitive functioning, decision making, attention and motivation (Cools et al., 2011, Grace, 2007, Nieoullon, 2002). Many functions under the influence of dopamine are altered with the progression of HAND (Abassi, 2017, Sacktor, 2018). Understanding biological factors affecting the state of dopamine in the brain may therefore provide further insight into the progression of HAND.

Catechol-O-methyltransferase (COMT) plays a role in metabolising dopamine by converting dopamine to inactive 3-methoxytyramine (Tenhunen et al., 1994). COMT is notably active in the prefrontal cortex, a brain region linked to cognition, executive function and memory (Tunbridge et al., 2013). Genetic variation in COMT modulates enzymatic activity and has been associated with changes in cortical dopamine (Tunbridge et al., 2013), which may affect behavioural and prefrontal cortex-dependent cognitive processes (McCane, 2018, Scheggia, 2018, Yavich, 2007).

A substitution of the amino acid valine (Val) with methionine (Met) at codon 158 (SNP rs4680) of the *COMT* gene has been associated with decreased COMT activity and slower metabolism of dopamine, resulting in a greater availability of dopamine at the synapse, and consequently, enhanced cognitive performance (Chen, 2004, Tunbridge, 2013). A neuroimaging study showed that altered *COMT* expression was linked to changes in prefrontal cortex activation and functional connectivity during working memory task performance, with *COMT* Val158 being associated with greater activation of the dorsolateral prefrontal cortex during task performance (Tunbridge et al., 2013). Variation in COMT activity, due to genetic alterations, in people living with HIV may therefore contribute toward altered functioning within neurocognitive domains such as executive function and memory (Yavich et al., 2007).

The dopamine transporter (DAT) is a protein necessary for the uptake of synaptic dopamine, with decreased DAT functioning leading to depleted intracellular levels of dopamine (Horn et al., 2013). DAT plays a critical role in the regulation of dopamine activity in subcortical brain structures by clearing released dopamine from the synapse to regulate the temporal and spatial availability of dopamine (Wang et al., 2004). The role of DAT in the clearance and availability of dopamine in the brain can be linked to cognitive function and behaviour, two of the key features of HAND (Chang, 2008, Horn, 2013). Positron emission tomography imaging shows that higher plasma viral load in patients with HAD correlated with a decreased level of the dopamine transporter (DAT) in the putamen (Wang et al., 2004). HAD was also associated with increased dopamine in the cerebrospinal fluid (Horn et al., 2013).

The *DAT* 10-repeat allele of a variable number of tandem repeats (VNTR) in exon 15 of the *DAT* gene is often associated with increased DAT expression (Heinz, 2000, Mill, 2002, VanNess et al., 2005) leading to increased DAT activity and dopamine reuptake and consequent decreased synaptic dopamine levels. While this variant is not associated with amino acid variation, it may alter mRNA stability and translational efficiency (Heinz, 2000). A study using single positron emission computed tomography in abstinent alcoholics found that the *DAT* 10/10 genotype resulted in a 22% increase in DAT protein availability compared to the 9/10 genotype (Heinz et al., 2000). An *in vitro* study on *DAT* expression and pharmacology also reported that homozygosity for the 10-repeat allele was associated with increased *DAT* expression and lower dopamine availability. The presence of a 9-repeat allele has been associated with higher dopamine availability and subsequently higher cortical activation during cognitive testing (VanNess, Owens and Kilts, 2005). Nevertheless, the *DAT* 10/10 genotype has also been linked to decreased *DAT* mRNA expression and a consequent increase in CSF dopamine in a cohort of South African and German participants with and without HIV (Horn et al., 2013). Genotype-associated differences in expression could influence HAND, with a study finding a significant reduction in available DAT in participants with HAD compared to seropositive controls without HAD (Wang et al., 2004). Dopamine transporter availability has been negatively associated with task errors during a neurocognitive card sorting test in healthy controls, with reduced striatal DAT availability associated with poorer task performance (Yen, 2015). Furthermore, reduced DAT availability in the putamen and caudate predicted poorer performance on multiple neuropsychological tests in participants with and without HIV (Chang et al., 2008).

### 1.4 Rationale and aim

South African women are at a particularly high risk for developing HAND, firstly because of the higher HIV prevalence in women living in South Africa, and secondly due to the high levels of childhood trauma experienced by South African women and the worsening effects of these experiences (Harrison, 2015, Spies, 2017). There is a need to identify biomarkers which will allow for the identification of individuals with HIV who are at higher risk for developing HAND, to facilitate the implementation of early intervention strategies to prevent the progression of HAND.

People living with HIV with a baseline diagnosis of asymptomatic neurocognitive impairment have a two- to six-fold increased risk of developing early mild neurocognitive disorders and HAD after several years (Grant et al., 2014). Biomarkers indicating the risk of conversion from asymptomatic neurocognitive impairment to more severe forms of HAND, such as HAD, would allow for intervention strategies to be implemented effectively and timeously. The identification of biomarkers associated with cognitive improvement would enable more accurate assessment of interventions and effectiveness of diagnostic neuropsychological tests (Saylor et al., 2016). At present, combined antiretroviral therapy is an option to delay the progression of HAND, but this is only effective in a subset of people living with HIV (Sacktor, 2018, Yuan and Kaul, 2019). The notable increased rate of progression of HAND in people living with HIV with childhood trauma remains a health concern in South Africa (Leserman, 2008, Spies, 2017).

The current exploratory study investigates the association between the *COMT* Val158Met polymorphism (rs4680) and the *DAT*-3′UTR VNTR and longitudinal neurocognitive function in a South African cohort of women living with and without HIV infection, with varying degrees of childhood trauma, in order to determine possible genetic and environmental risk factors for HAND.

## 2 Methods

### 2.1 Ethical considerations and participant recruitment

This study was approved by the ethics committee of Stellenbosch University, South Africa [N07/07/153] and written informed consent was obtained from all participants. Participants for this study were previously recruited by researchers from the Department of Psychiatry, Stellenbosch University, for a larger study titled “Biological Endophenotypes of HIV and Childhood Trauma: A Genetics, Cognitive and Imaging Study.” Participant recruitment and the assessments of childhood trauma and neurocognitive function were conducted by trained personnel and in accordance with the Declaration of Helsinki.

Participants were recruited directly from the community or through community health care facilities around the Cape Metropole of South Africa. The sample consisted of 49 HIV-negative women and 64 women living with HIV. Participants were included in the study if they were between 18 and 65 years of age; able to undergo neuropsychological testing; able to read and write in English, Afrikaans or isiXhosa (an African indigenous language predominantly spoken in the Western Cape) at 5th grade level; and were willing and able to provide written informed consent. Exclusion criteria were current or past history of psychiatric disorders, including current alcohol and/or drug use disorder, as assessed using the Mini-International Neuropsychiatric Interview-Plus (MINI-Plus); significant previous head injury; current seizure disorders; a history of CNS infections or neoplasms; hepatitis B or C positive status, or current use (within the last month) of any psychotropic medication.

### 2.2 Collection of demographic and clinical data

Demographic data, such as age, marital status, self-reported ethnicity, years of education, and employment status were captured. The HIV status of participants was tested and confirmed using an enzyme-linked immunosorbent assay. HIV serology testing was performed on all HIV-negative controls to confirm HIV negative status at the first screening visit, roughly one week prior to neurocognitive testing. The same process was repeated at the 1-year follow-up, where all participants were tested again to confirm HIV status and identify seroconversions. Participants in the HIV-positive group were tested at their primary health care facility at the time of voluntary counselling and testing. Virology measures such as CD-4 and CD-8 lymphocyte count and viral load were obtained from blood samples at baseline and 1-year follow-up.

### 2.3 Childhood trauma questionnaire

Childhood trauma exposure was assessed using the Childhood Trauma Questionnaire Short Form (CTQ-SF), a 28-item self-report questionnaire, which retrospectively assesses emotional, physical and sexual abuse, and emotional and physical neglect in early life (before the age of 18) (Bernstein et al., 2003). Each of the 5 trauma sub-scales consist of 5 items with a score ranging from 5 to 25. These scores are combined to give a total CTQ-SF score. Higher total scores are indicative of higher levels of childhood trauma with a score of less than 31 indicating little to no experience of trauma. For this study, individuals were grouped as either having experienced childhood trauma of at least mild severity (indicated by a score of 41 or more) or not (indicated by a score of less than 31) (Bernstein et al., 2003).

### 2.4 Neurocognitive measurement

Neurocognitive function was assessed using the HIV Neurobehavioral Research Centre International Neurobehavioral battery for detecting and diagnosing HAND (Heaton et al., 2008). This battery assesses seven domains of cognitive function: learning, delayed recall, processing speed, attention/working memory, executive function, verbal fluency and motor ability (Heaton, 2008, Spies, 2017) (Table 1). The tests were translated into Afrikaans and isiXhosa, and were administered according to the participants’ self-reported home language. Appropriate modifications to test instructions and stimuli were made to fit a South African cultural context (Spies et al., 2017). Raw test scores were subjected to regression analyses using age and education as predictor variables and test scores as the outcome. The resultant residuals were used to calculate studentized residuals, which were then summed to create a domain Z-score. The mean of the seven domain Z-scores was calculated to provide an age- and education-adjusted composite global cognitive score.

**Table 1:** HNRC Neuropsychological Test battery.

Neuropsychological domain	Neuropsychological test
Speed of information processing	WAIS-III Digit Symbol WAIS-III Symbol Search Trail Making Test Part A
Attention / Working memory	Paced Auditory Serial Addition Test WMS-III Spatial Span
Executive functioning	Wisconsin Card Sorting Test – computer version Colour Trails 1 and 2Stroop Colour Word Test Halstead Category Test – computer version
Learning and delated recall	Hopkins Verbal Learning Test – Revised (HVLT-R) Brief Visuospatial Memory Test – Revised (BVMT-R)
Language	Controlled Word Association Test Category Fluency (animal fluency and action fluency)
Motor	Grooved Pegboard Test (both hands)
Screening for effort	Hiscock Digit Memory Test

(Heaton et al., 2008)

### 2.5 Polymerase chain reaction

DNA extracted from whole blood was used as a template in polymerase chain reactions (PCR). The following primers were used for the PCR (Integrated DNA Technologies, Coralville, IA):

*COMT* Forward: 5′*TCGAGATCAACCCCGACTGT*3′.

*COMT* Reverse: 5′*TGGGTTTTCAGTGAACGTGGT*3′.

*DAT* Forward: 5′*ATGGGGGTCCTGGTATGTCT*3′.

*DAT* Reverse: 5′*GGCACGCACCTGAGAGAAAT*3′.

The *COMT* Val158Met PCR was performed in 10 µl reactions containing 5 µl KAPA2G Robust HotStart ReadyMix (Kapa Biosystems, Wilmington, MA), 3 µl PCR grade H_2_O, 0.5 µl 10 mM *COMT* forward primer, 0.5 µl 10 mM *COMT* reverse primer and 1 µl template DNA with a concentration of between 30 ng/µl to 100 ng/µl. The PCR conditions for the *COMT* Val158Met polymorphism were as follows: initial denaturing at 92 °C for 5 min; 35 cycles of denaturation at 92 °C for 30 s, annealing at 62 °C for 30 s, extension at 72 °C for 30 s, and a final extension step at 72 °C for 5 min. Product size was reported to be 244 bp (Saravani, Galavi and Lotfian Sargazi, 2017).

The *DAT* VNTR PCR was performed in 10 µl reactions containing 5 µl KAPA2G Robust HotStart ReadyMix, 3 µl PCR grade H_2_O, 0.5 µl 10 mM *DAT* forward primer, 0.5 µl 10 mM *DAT* reverse primer and 1 µl template DNA with a concentration of between 30 ng/µl to 100 ng/µl. The following PCR conditions were used for the amplification of the *DAT* 40 bp-VNTR: initial denaturation at 95 °C for 10 min; 35 cycles of denaturation at 93 °C for 1 min, annealing at 60 °C for 30 s, extension at 72 °C for 1 min, and a final extension at 72 °C for 10 min. Product size ranged from 311 base pairs to 596 base pairs for the repeat. All PCR experiments were carried out using the Applied Biosystems 2720 Thermal Cyler (Applied Biosystems, Foster City, CA).

### 2.6 COMT restriction enzyme digest

A restriction enzyme digestion was performed to cleave the *COMT* PCR product at the SNP site. This was performed in 10 µl reactions containing 2.8 µl PCR-grade H_2_O; 0.8 µl CutSmart10x Buffer (New England Biolabs, Ipswitch, MA); 0.4 µl *Nla*III; and 6 µl *COMT* PCR product. The *COMT* Val158 homozygotes had fragment sizes of 86 and 23 base pairs, the Met158 homozygotes had fragment sizes of 68 and 18 base pairs, and the Val158Met heterozygotes had fragment sizes of 86, 68, 23 and 18 base pairs in length (Malhotra et al., 2002).

### 2.7 Gel electrophoresis

A 15% acrylamide gel was used to visualise the *COMT* Val158Met polymorphism using 40% acrylamide solution (Sigma-Aldrich, St. Louis, MO), tetramethylethylenediamine (Sigma-Aldrich) and ammonium persulfate (Sigma-Alrich). Restriction enzyme digest products were loaded with 3 µl Gel Loading Dye Purple (6x) (New England Biolabs) for each 5 µl sample of restriction digest product used. A GeneRuler Low Molecular Weight (25 bp) DNA Ladder (New England Biolabs) was used as a standard marker in a 1:12 dilution. Electrophoresis was done at 120 V for approximately 4 h using the BioRad PowerPac Basic system. The gel was then stained for 30 min with 3 µl CondaSafe (Condalab, Madrid, Spain) in 50 ml 1X tris borate EDTA running buffer before visualisation using the BioRad Universal Hood III UV light (BioRad Laboratories, Hercules, CA).

The *DAT* 40 bp VNTR was visualised using a 1.5% agarose gel (SeaKem LE, Lonza, Basel, Switzerland). PCR products were loaded on to the gel with 3 µl Gel Loading Dye Purple (6x). Electrophoresis was conducted for 2.5 h at 70 V using the BioRad PowerPac Basic system. A GeneRuler 50 bp DNA ladder was used as a standard marker in a 1:12 dilution (New England Biolabs). The gel was pre-stained with 3 µl CondaSafe and visualised using the BioRad Universal Hood III UV light.

**Fig. 1:**
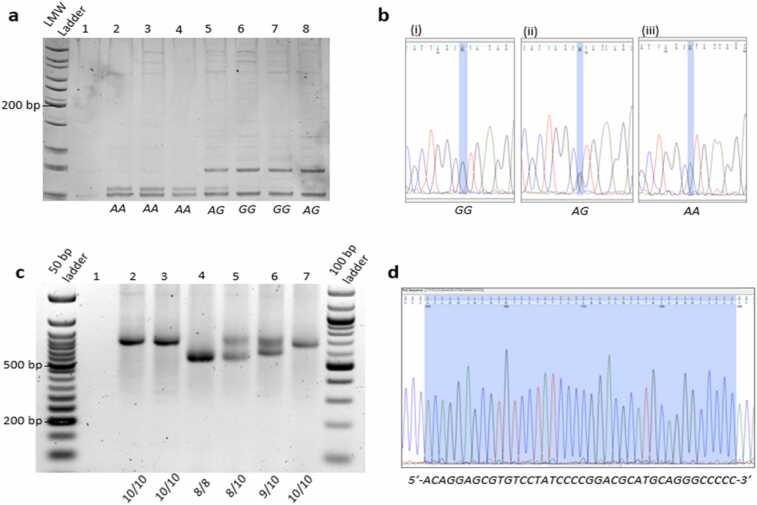
**:** a) *COMT* genotyping using a 15% acrylamide gel. Samples were genotyped for the *COMT* rs4680 variant using acrylamide gel electrophoresis. The different genotypes can be clearly seen on the gel. *GG* describes the *Val/Val* genotype; *AG* describes the *Val/Met* genotype; and *GG* describes the *Met/Me*t genotype. A 25 bp molecular marker is used as a reference. b) *COMT* Sanger sequencing for COMT rs4680. (i) a homozygous *GG (Val/Val*) genotype; (ii) a heterozygous *AG (Val/Met)* genotype; (iii) a homozygous *AA (Met/Met)* genotype using Sanger sequencing. c) *DAT* genotyping using a 1.5% agarose gel. *DAT* 40 bp VNTR variants were genotyped on an agarose gel stained with Condasafe. The electrophoresis was carried out at 70 V for 2.5 h. Lane 4 = */* ; lanes 5 and 6 = */10; and lanes 2, 3, and 7 – 10/10 genotype. Lane 1 represents the negative control. d) *DAT*-VNTR sequence chromatogram using Sanger sequencing.

The use of agarose gels to determine *DAT* VNTR genotype may be influenced by subjectivity and human error. To minimise the effects of this potential bias, homozygous samples were selected for Sanger sequencing. Homozygous samples were used as a reference alongside the DNA ladder to provide a more accurate measure of genotype (Fig. 1d).

### 2.8 Sanger sequencing

For both *COMT* and *DAT*, bi-directional Sanger sequencing was used to validate genotypes obtained using acrylamide and agarose gel electrophoresis, respectively. Twenty-one *COMT* Val158Met PCR and 16 *DAT*-VNTR PCR amplicons were sequenced. Samples were chosen to verify a wide range of genotypes according to their position on the gel. The PCR was performed prior to sending the samples for sequencing as described above. Post-PCR clean-up and Sanger sequencing procedures were conducted at the Central Analytical Facility SU, South Africa.

### 2.9 Data analysis

As the results of a Shapiro-Wilk W test indicated that CTQ-SF scores were not normally distributed, inverse sin-transformed scores were included in statistical models. A student’s *t*-test was used to determine group differences (HIV status) for normally distributed data, and a Wilcoxon test was used for non-parametric data. Linear regression models were used to determine associations between HIV status, childhood trauma and genotype, on the outcome variable (an age and education adjusted global cognitive Z-score). To assess for changes in cognitive scores, baseline scores were included as a predictor variable. Additive and interaction regression models were used to determine whether any interactions were associated with changes in the outcome variable. Analysis of variance was used to assess whether including interaction effects between predictor variables in models explained significantly more of the variation in the outcome. All statistical analyses were performed in R (version 3.6.0) using packages *HardyWeinberg* to test for Hardy-Weinberg equilibrium, *tidyverse* and *dplyr* for data analysis and *ggplot2* to construct graphical models. Normally distributed data are reported as mean ± SD, whereas non-parametric data are reported using the median and interquartile range. Associations were noted as significant when the *p*-value was less than or equal to 0.05 and associations were regarded as trend-level when the p-value was more than 0.05 but less than 0.1.

## 3 Results

### 3.1 Cohort description

The cohort consisted of 113 women between the ages of 18 and 50 years with a mean age of 33 ± 7.77 years at baseline. Ninety-three percent of participants reported isiXhosa as their home language. The average level of education was 10.35 ± 1.77 years.

### 3.2 Neuropsychological assessments according to HIV status

HIV positive and negative groups differed significantly in their cognitive performance at baseline and one-year follow-up time points, with worse cognitive performance in the HIV-positive group (p = 2.041 ×10^−05^) at 1 year (Table 2). The HIV-positive group also reported significantly higher exposure to childhood trauma (p = 7.758 ×10^−40^) with 78% of HIV-positive women having experienced any form of trauma before the age of 18 years compared to 36.7% of HIV-negative participants who reported experiencing any form of childhood trauma before 18 years of age (Table 2).

**Table 2:** Neuropsychological assessments according to HIV status.

Variable	HIV-positive (*n* = 64)	HIV-negative (*n* = 49)	*P*
Mean age in years ± SD	30.86 ± 8.5	34.64 ± 6.76	0.051
Median education in years ± IQR	11 ± 2	11 ± 1	7.835 × 10^−7^
Median CTQ-SF score ± IQR	63.5 ± 31.5	35.0 ± 17.0	7.758 × 10^−40^
Median baseline global cognitive score ± IQR	-0.086 ± 0.593	0.084 ± 0.612	1.011 × 10^−14^
Mean follow-up global cognitive score±	-0.147 ± 0.513	0.199 ± 0.589	1.295 × 10^−13^

CTQ-SF = childhood trauma questionnaire – short form; *p* = p-value; SD = standard deviation; SD = standard deviation; IQR = interquartile range

### 3.3 *DAT* and *COMT* genotype according to HIV status

One-hundred and thirteen samples were genotyped for the *DAT-*VNTR and *COMT* variant of interest (rs4680). Due to the inability to clearly identify the genotype for one *COMT* and one *DAT* sample after using both electrophoresis and Sanger sequencing methods, these two samples, belonging to different participants, were excluded from analysis. This resulted in 112 samples being used for each analysis. Both *COMT* (p = 0.200) and *DAT* (p = 0.378) genotype groupings were in Hardy-Weinberg equilibrium.

For *COMT* analysis, participants were grouped and analysed according to their presence or absence of an *A* allele (Table 3). In this cohort, 61 participants were homozygous for the Val allele (*GG*) and therefore absent of an A allele and 51 participants had at least one A allele. Of these 51 participants, 39 participants were heterozygous for the Val158Met SNP (*GA*), and 12 participants were homozygous for the Met allele (*AA*).

**Table 3:** Number of individuals (%) per *COMT Val158Met and DAT-VNTR* genotype group in women living with HIV (n = 64) and without HIV (N = 48).

*COMT* Genotype	HIV-positive N= 64	HIV-negative N= 48	X^2^	p-value
*AA*	5 (7.8%)	7 (14.6%)	0.702	0.402
*AG*	24 (37.5%)	15 (31.3%)	0.237	0.626
*GG*	35 (54.7%)	26 (54.2%)	3.621 × 10^−31^	1
*DAT* Genotype				
* /*	16 (25%)	9 (18.8%)	0.310	0.577
* /10	27 (42.2%)	23 (47.9%)	0.169	0.681
10/10	21 (32.8%)	16 (33.3%)	6.872 × 10^−31^	1

This table reports the number of individuals per *COMT* and *DAT* genotype according to HIV status. For *DAT* genotype, * /* refers to genotype absent of a *DAT* 10-repeat; * /10 refers to genotype heterozygous for a single *DAT* 10-repeat; 10/10 refers to homozygosity for the 10-repeat allele. P-value indicates the difference in HIV status of participants for each genotype.

*DAT* VNTR genotypes were grouped and analysed according to the presence or absence of a 10-repeat allele (Caldú et al., 2007) (Table 3). In this cohort, 25 individuals had no 10-repeat allele present (denoted as */*) and 87 participants had at least a single 10-repeat allele. Of these 87 participants, 50 individuals were heterozygous for a 10-repeat allele (*/10), and 37 participants were homozygous for the 10-repeat allele (10/10).

### 3.4 HIV and childhood trauma interaction on cognitive scores

HIV-seropositivity was associated with significantly lower follow-up global cognitive scores (*p* = 0.001). Childhood trauma was inversely associated with global cognitive scores at baseline (*p* = 0.050) and follow-up (*p* = 0.018). The interaction between childhood trauma and HIV status showed a trend toward significant association with global cognitive scores in longitudinal models (p = 0.065), whereby HIV-seropositivity and the experience of childhood trauma indicated a risk for poorer global cognitive function. Including the *CT* *x* *HIV* interaction in regression models revealed a trend towards explaining more of the variance in follow-up cognitive scores (p = 0.062).

### 3.5 Childhood trauma and dopaminergic genetic variation interaction on cognitive scores

**Fig. 2:**
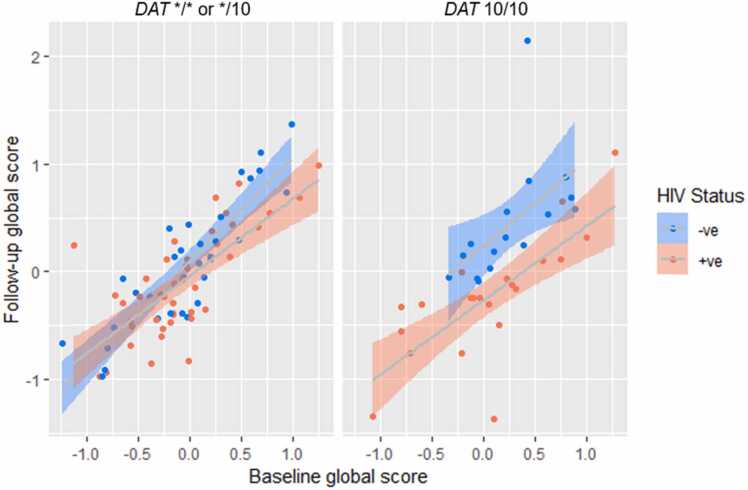
Scatter plot showing the interaction between *DAT* 10/10 genotype and HIV status significantly associated with follow-up global cognitive scores (HNRC battery). HIV seropositivity and *DAT* 10-repeat homozygosity was associated with significantly lower cognitive scores in longitudinal models (p = 0.010). The regression model was generated using age and education adjusted z-scores and data is displayed as raw data points. The figure shows a shaded 95% confidence interval with trend lines.

**Fig. 3:**
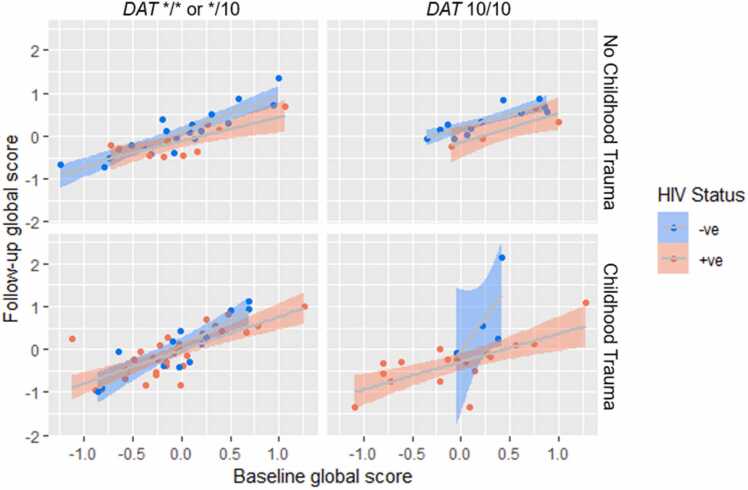
Scatter plot showing the relationship between *DAT* 10/10, childhood trauma (CTQ-SF) and HIV status trend towards association with longitudinal cognitive scores (p = 0.08). Including the interaction between DAT 10-repeat homozygosity, childhood trauma and HIV status explained significantly more of the variation in follow-up cognitive scores (p = 0.008). Childhood trauma is displayed categorically with scores less than or equal to 31 being categorized as “No Childhood Trauma” group and scores more than or equal to 41 being categorized as the “Childhood Trauma” group. The regression model was generated using age and education adjusted z-scores and data is displayed as raw data points. The figure shows a shaded 95% confidence interval with trend lines.

In the analysis of women living with HIV, *DAT* 10-repeat homozygosity was associated with significantly lower cognitive scores in longitudinal models (*p* = 0.023). The interaction of the *DAT* 10-repeat homozygous genotype and HIV status was significantly associated with cognitive scores in longitudinal models (*p* = 0.010), and including this interaction explained significantly more of the variance seen in cognitive scores (*p* = 0.009) (Fig. 2). The interaction of *DAT* 10-repeat homozygosity, childhood trauma and HIV status produced a trend-level association with longitudinal cognitive scores (*p* = 0.090), and including this interaction explained significantly more of the variance (*p* = 0.008) (Fig. 3).

**Fig. 4:**
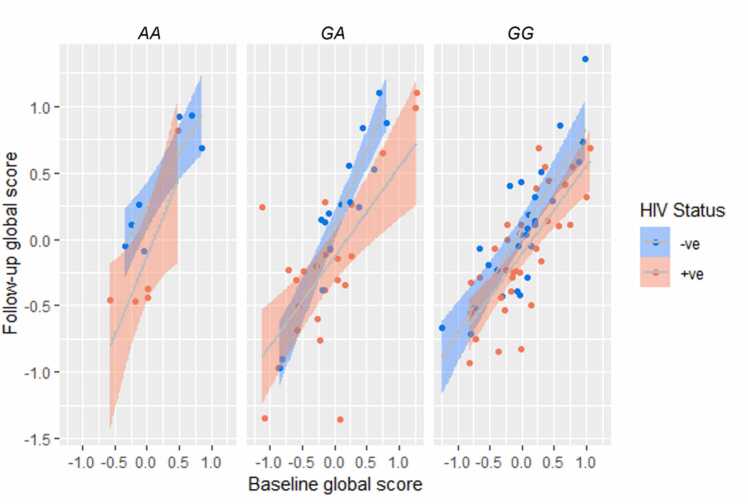
Scatter plot showing the relationship between COMT genotype and HIV status with longitudinal cognitive scores (p = 0.5105). The regression model was generated using age and education adjusted z scores, and data is displayed as raw data points. The figure shows a shaded 95% confidence interval with trend lines.

No significant associations with *COMT* genotypes were identified. No significant association between *COMT* rs4680 A-allele genotype group and longitudinal cognitive scores were noted (p = 0.386), nor was the interaction of *COMT* genotype, HIV status and childhood trauma significant in predicting longitudinal cognitive scores (p = 0.845). The interaction of *COMT* and HIV status did not predict global longitudinal cognitive scores (p = 0.509); neither did the interaction of *COMT* with childhood trauma scores (p = 0.742) (Fig. 4).

## 4 Discussion

This study is an exploratory investigation of the association of *DAT* and *COMT* genotypic variants and cognitive function over time in women living with HIV. Childhood trauma is highly prevalent in the South African setting, and is also known to affect cognitive function (Jewkes et al., 2001, Leserman, 2008, Spies, 2017). In light of this, we investigated whether the interaction between childhood trauma and genotype influenced cognitive function in a cohort of women living with and without HIV in South Africa. Our findings suggest that the interaction between childhood trauma and genetic variation in the *DAT* 40 bp VNTR may contribute toward the aetiology of HAND. This study found significantly lower cognitive scores in longitudinal models for the participants with the *DAT* 10/10 genotype compared to the other *DAT* genotype groups. The homozygous 10-repeat genotype has been associated with reduced *DAT* mRNA expression, and higher CSF dopamine levels (Horn et al., 2013). This has in turn been associated with worse cognitive function (Wang et al., 2004). A positron emission tomography study in participants with HIV found evidence for decreased DAT levels in those with vs. without HAD (Wang et al. 2004), further corroborating our results. In opposition to our hypothesis of reduced DAT linked to HAND, an increase in the expression of DAT mRNA has been associated with the *DAT* VNTR 10/10 genotype (Heinz et al., 2000; Mill et al., 2002; VanNess, Owens and Kilts, 2005).

With respect to *COMT,* we did not observe any genotype effect on cognitive scores. This is in keeping with the results of two previous studies (Horn, 2017, Levine, 2012) investigating the role of *COMT* genetic variation and neurocognitive function in the context of HIV. Nevertheless, multiple studies have found evidence for an association between the Val allele and worse cognitive function (Bousman, 2010, Saloner, 2019, Sundermann, 2015). As this allele corresponds with more rapid metabolism and thus lower dopamine bioavailability, this would contradict the inverse relationship between dopaminergic signalling and cognitive function suggested by our and others *DAT* 10/10 results (Horn et al., 2013), as well as the neuroimaging findings of Wang et al. (2004). It is worth noting that in two of these studies, the genotype effect of *COMT* Val158Met was only significant in interaction with other risk factors, namely cardiometabolic risk (Saloner et al., 2019) and methamphetamine dependence (Bousman et al., 2010), neither of which were assessed in the current study. Furthermore, the role of COMT in neurotransmission also extends to other catecholamines such as norepinephrine (Reenilä and Männistö, 2001). It is thus possible that previous findings of associations between the Val allele and cognitive function may be driven by noradrenergic rather than dopaminergic effects. Ultimately, dopaminergic signalling is a complex process, with multiple genetic variants likely having an effect. The effect of *DAT* and *COMT* variation on dopamine transmission and cognitive function may be better understood using more in-depth genetic studies where the influence of multiple dopamine-related genetic variants can be accounted for. Along a similar line, more detailed genetic investigations combined with neuroimaging techniques would assist in determining the role of genetically driven variation in dopaminergic signalling in neurocognition.

Sixty percent of women in the cohort reported experiencing childhood trauma corresponding with the high proportion of childhood trauma in South Africa reported in existing literature (Choi et al., 2015). As expected, women living with HIV had lower global cognitive scores at baseline and follow-up and experienced significantly greater cognitive decline than the HIV-negative group. This may be explained by the effect of HIV-induced neuronal damage and its possiblerole in mediating cognition (Everall, 1999, de Pablos, 2014).

This study demonstrates the negative effects that childhood trauma may have on cognitive function in people living with HIV, in line with previous literature (Denckla et al., 2017, Spies, 2017, Spies, 2016). The effect on cognition may be explained by compromised cellular immunity induced by psychosocial stressors, such as childhood trauma, leading to the faster progression of HIV and its neurotoxic effects (Leserman, 2003). Future studies in larger cohorts are warranted to verify these results.

We identified no significant association between *DAT* or *COMT* genotype and HIV status using a Pearson’s chi-squared test with Yates continuity correction, contrary to findings in another South African cohort by Horn et al. (2013) of an association between *DAT* 10/10 and increased risk for HIV. The current study is specific to South African women of Xhosa ethnicity, whereas the sample of Horn et al. (2013) was a cohort of male and female South Africans with Xhosa or mixed ethnicities, as well as German participants. In addition, the increased prevalence of HIV associated with *DAT* 10/10 is thought to be behaviour related, with the 10/10 genotype being associated with higher impulsivity, hyperactivity, and weaker inhibition control (Congdon et al., 2008, Cornish, 2005, Roman, 2001). These behavioural associations with *DAT* VNTR genotype are mostly present in male cohorts (Cornish et al., 2005), not representative of our sample, or have small effects on outcome measures, usually in interaction with other genes such as *DRD4*, which was not assessed in the current study (Congdon et al., 2008, Roman, 2001). This effect may therefore be sex-specific, or may describe an interaction that was not examined in the present study.

Although precautions have been taken to address study limitations as far as possible, a few limitations do warrant mention. First, a larger sample would improve statistical power facilitating more accurate identification of smaller size effects. Second, the CTQ-SF is a retrospective self-report assessment of childhood trauma. As such, data may be influenced by recall bias. In addition, our data does not account for trauma experienced in adulthood and does not distinguish between single and repeated events. Third, this research only included women. Although women are disproportionately affected by HIV in South Africa and are therefore more vulnerable to developing HAND, it would be beneficial to assess the relative contributions of genetic variation and childhood trauma in South African men as well, to better understand sex differences.

Despite these limitations, this study has notable strengths. Our study was successful in determining the association between dopamine-related genetic variation and childhood trauma as risk factors for the progression of HAND in a South African population. Using a longitudinal approach, we observed HIV-induced cognitive decline. We conducted detailed assessments using a well-validated neuropsychological battery and controlled for confounding variables such as age and education. Since both HIV and childhood trauma are highly prevalent amongst South African women, understanding the contribution of risk factors and identifying biomarkers of rapid cognitive decline may facilitate the development of promising intervention strategies.

There is a need for replication in larger cohorts and investigation of different types of childhood trauma to better understand the extent to which childhood trauma and genetic variation contribute to cognitive decline in people living with HIV. Although our study confirms decreased neurocognitive function in participants with HIV, it is important to note that the effects of disease severity and neuropathology have not been studied in association with the neurocognitive features of HAND. Virology markers such as CD4 lymphocyte count and viral load obtained during baseline and follow up time points from the CSF may provide a better understanding of disease progression and HAND. Future studies may include an exploration of epistatic interactions between *COMT* and *DAT* in the development of HAND, or how other neurotransmitter-related genes are associated with the development of HAND. Additional research is also needed to ascertain the mechanisms underlying the interactions described. The effects of HIV, environmental factors, and biological factors such as genetic variants affecting dopamine regulation should be researched holistically to better understand the reduced cognitive functioning associated with HAND.

In conclusion, this study provides preliminary evidence for genetic and environmental influences on cognition, which may prove beneficial in identifying individuals at a greater risk for developing HAND. Insight into the pathways that govern observed interactions will facilitate a better global understanding of their effects on long-term cognitive functioning.

## Funding sources

This study was supported by a Centres for AIDS research (CFAR) grant awarded to SS (“Biological Endophenotypes of HIV and Childhood Trauma: A Genetics, Cognitive and Imaging study”), grant number P30 AI036214. AR, JSW, PS, GS, SMJH and SS are supported by the South African Research Chair in PTSD from the Department of Science and Technology and the National Research Foundation. Research reported in this publication was partly supported by the South African Medical Research Council. Research funding was also received from The Harry Crossley Foundation.

## Competing interests

None.

## Summary compliance with ethical standards

This study was approved by the ethics committee of Stellenbosch University, South Africa (ethics reference number: N07/07/153] under a larger study titled “Biological Endophenotypes of HIV and Childhood Trauma: A Genetics, Cognitive and Imaging Study.”

## References

[bib1] Abassi M. (2017). Cerebrospinal fluid biomarkers and HIV-associated neurocognitive disorders in HIV-infected individuals in Rakai, Uganda. J. Neurovirology.

[bib2] Andersson N., Cockcroft A., Shea B. (2008). Gender-based violence and HIV: relevance for HIV prevention in hyperendemic countries of southern Africa. AIDS.

[bib3] Antinori A. (2007). Updated research nosology for HIV-associated neurocognitive disorders. Neurology.

[bib4] Atluri V.S.R. (2015). Effect of human immunodeficiency virus on blood-brain barrier integrity and function: an update. Front. Cell. Neurosci..

[bib5] Berke J. (2018). What does dopamine mean?. Nat. Neurosci..

[bib6] Bernstein D.P. (2003). Development and validation of a brief screening version of the childhood trauma questionnaire. Child Abus. Negl..

[bib7] Bousman C.A. (2010). Impact of COMT Val158Met on executive functioning in the context of HIV and methamphetamine. Neurobehav. HIV Med..

[bib8] Caldú X. (2007). Impact of the COMT Val108/158 Met and DAT genotypes on prefrontal function in healthy subjects. NeuroImage.

[bib9] Chaganti J.R. (2017). Functional connectivity in virally suppressed patients with hiv-associated neurocognitive disorder: a resting-state analysis. Am. J. Neuroradiol..

[bib10] Chang L. (2008). Decreased brain dopamine transporters are related to cognitive deficits in HIV patients with or without cocaine abuse. NeuroImage.

[bib11] Chen J. (2004). Functional analysis of genetic variation in catechol-O-methyltransferase (COMT): effects on mRNA, protein, and enzyme activity in postmortem human brain. Am. J. Hum. Gen..

[bib12] Choi K.W. (2015). Maladaptive coping mediates the influence of childhood trauma on depression and PTSD among pregnant women in South Africa. Arch. Women’s Mental Health.

[bib13] Congdon E., Lesch K.P., Canli T. (2008). Analysis of DRD4 and DAT polymorphisms and behavioral inhibition in healthy adults: implications for impulsivity. Am. J. Med. Gen..

[bib14] Cools R., Nakamura K., Daw N.D. (2011). Serotonin and dopamine: unifying affective, activational, and decision functions. Neuropsychopharmacol. Official Publication Am. College Neuropsychopharmacol..

[bib15] Cornish K.M. (2005). Association of the dopamine transporter (DAT1) 10/10-repeat genotype with ADHD symptoms and response inhibition in a general population sample. Mol. Psychiatry.

[bib16] Cunningham P.H. (2000). Evidence for independent development of resistance to HIV-1 reverse transcriptase inhibitors in the cerebrospinal fluid. AIDS.

[bib17] Deiss R. (2019). Posttraumatic stress disorder and neurocognitive impairment in a U.S. military cohort of persons living with HIV. Psychiatry Interpers. Biol. Process..

[bib18] Denckla, C.A. et al. (2017) Associations between neurocognitive functioning and social and occupational resilience among South African women exposed to childhood trauma. European Journal of Psychotraumatology. [online]. 8 (1). Available from: https://www.ncbi.nlm.nih.gov/pmc/articles/PMC5687801/ [Accessed 18 October 2019]. 10.1080/20008198.2017.1394146 PMC5687801 29163865

[bib19] Everall I.P. (1999). Cortical synaptic density is reduced in mild to moderate human immunodeficiency virus neurocognitive disorder. Brain Pathol..

[bib20] Grace A.A. (2007). ‘Regulation of firing of dopaminergic neurons and control of goal-directed behaviors’. Trends Neurosci..

[bib21] Grant I. (2014). Asymptomatic HIV-associated neurocognitive impairment increases risk for symptomatic decline. Neurol..

[bib22] Harrison A. (2015). Sustained High HIV Incidence in Young Women in Southern Africa: Social, Behavioral and Structural Factors and Emerging Intervention Approaches. Curr. HIV/AIDS Rep..

[bib23] Heaton R.K. (2008). Neurobehavioral effects of human immunodeficiency virus infection among former plasma donors in rural China. J. Neurovirol..

[bib24] Heaton R.K. (2011). HIV-associated neurocognitive disorders before and during the era of combination antiretroviral therapy: differences in rates, nature, and predictors. Journal of NeuroVirology. [online]..

[bib25] Heinz A. (2000). Genotype influences in vivo dopamine transporter availability in human striatum. Neuropsychopharmacology: Official Publication of the American College of Neuropsychopharmacology.

[bib26] Hong S., Banks W.A. (2015). Role of the immune system in HIV-associated neuroinflammation and neurocognitive implications. Brain Behav. Immun..

[bib27] Horn A. (2013). Increases in CSF dopamine in HIV patients are due to the dopamine transporter 10/10-repeat allele which is more frequent in HIV-infected individuals. J. Neural Transm..

[bib28] Horn A. (2017). The dopamine-related polymorphisms BDNF, COMT, DRD2, DRD3, and DRD4 are not linked with changes in CSF dopamine levels and frequency of HIV infection. J. Neural Transm..

[bib29] Jewkes R. (2001). Prevalence of emotional, physical and sexual abuse of women in three South African provinces. South Afr. Med. J. Suid Afrikaanse Tydskrif Vir Geneeskunde.

[bib30] Jewkes R. (2002). Rape of girls in South Africa. Lancet.

[bib31] Kalichman S.C., Simbayi L.C. (2004). Sexual assault history and risks for sexually transmitted infections among women in an African township in Cape Town, South Africa. AIDS care.

[bib32] Khoury M.N. (2013). CSF Viral Escape in a patient with HIV-Associated Neurocognitive Disorder. J. Neurovirol..

[bib33] Leserman J. (2003). HIV disease progression: depression, stress, and possible mechanisms. Biol. Psychiatry.

[bib34] Leserman J. (2008). Role of depression, stress, and trauma in HIV disease progression. Psychosom. Med..

[bib35] Levine A.J. (2012). Functional polymorphisms in dopamine-related genes: effect on neurocognitive functioning in HIV+ adults. J. Clin. Exp. Neuropsychol..

[bib36] Malhotra A.K. (2002). A functional polymorphism in the COMT gene and performance on a test of prefrontal cognition. Am. J. Psychiatry.

[bib37] Maschke M. (2000). Incidence and prevalence of neurological disorders associated with HIV since the introduction of highly active antiretroviral therapy (HAART). J. Neurol. Neurosurg. Psychiatry.

[bib38] McCane A.M. (2018). Differential COMT expression and behavioral effects of COMT inhibition in male and female Wistar and alcohol preferring rats. Alcohol.

[bib39] McCutcheon R.A. (2019). Mesolimbic dopamine function is related to salience network connectivity: an integrative positron emission tomography and magnetic resonance study. Biol. Psychiatry.

[bib40] Mill J. (2002). Expression of the dopamine transporter gene is regulated by the 3’ UTR VNTR: Evidence from brain and lymphocytes using quantitative RT-PCR. American Journal of Medical Genetics. [online]..

[bib41] Nieoullon A. (2002). Dopamine and the regulation of cognition and attention. Prog. Neurobiol..

[bib42] Nightingale S. (2014). Controversies in HIV-associated neurocognitive disorders. Lancet. Neurol..

[bib43] de Pablos R.M. (2014). Chronic stress enhances microglia activation and exacerbates death of nigral dopaminergic neurons under conditions of inflammation. J. Neuroinflamm..

[bib44] Pukay-Martin N.D. (2003). The relationship between stressful life events and cognitive function in HIV-infected men. J. Neuropsychiatry Clinic. Neurosci..

[bib45] Reenilä I., Männistö P.T. (2001). Catecholamine metabolism in the brain by membrane-bound and soluble catechol-o-methyltransferase (COMT) estimated by enzyme kinetic values. Med. Hypotheses.

[bib46] Roman T. (2001). Attention-deficit hyperactivity disorder: a study of association with both the dopamine transporter gene and the dopamine D4 receptor gene. Am. J. Med. Gen..

[bib47] Sacktor N. (2018). Changing clinical phenotypes of HIV-associated neurocognitive disorders. J. Neurovirol..

[bib48] Saloner R. (2019). COMT Val158Met polymorphism, cardiometabolic risk, and nadir CD4 synergistically increase risk of neurocognitive impairment in men living with HIV. J. Acquir. Immune Defic. Syndr..

[bib49] Saravani R., Galavi H.R., Lotfian Sargazi M. (2017). Catechol-O-Methyltransferase (COMT) gene (Val158Met) and brain-derived neurotropic factor (BDNF) (Val66Met) genes polymorphism in schizophrenia: a case-control study. Iran. J. Psychiatry.

[bib50] Saylor D. (2016). HIV-associated neurocognitive disorder--pathogenesis and prospects for treatment. Nat. Rev. Neurol..

[bib51] Scheggia D. (2018). Remote memories are enhanced by COMT activity through dysregulation of the endocannabinoid system in the prefrontal cortex. Mol. Psychiatry.

[bib52] Spies G. (2017). Changes in cognitive function in women with HIV infection and early life stress. AIDS Care.

[bib53] Spies G. (2016). Effects of HIV and childhood trauma on brain morphometry and neurocognitive function. J. Neurovirology.

[bib54] Sundermann E.E. (2015). Genetic predictor of working memory and prefrontal function in women with HIV. J. Neurovirology.

[bib55] Tenhunen J. (1994). Genomic organization of the human catechol O-methyltransferase gene and its expression from two distinct promoters. Euro. J. Biochem..

[bib56] Thomas J.B. (2013). Pathways to neurodegeneration: effects of HIV and aging on resting-state functional connectivity. Neurology.

[bib57] Tunbridge E.M. (2013). Catechol-O-methyltransferase (COMT) influences the connectivity of the prefrontal cortex at rest. NeuroImage.

[bib58] Turner H.A., Finkelhor D., Ormrod R. (2010). Poly-Victimization in a National Sample of Children and Youth. Am. J. Prev. Med. [online]..

[bib59] VanNess S.H., Owens M.J., Kilts C.D. (2005). The variable number of tandem repeats element in DAT1 regulates in vitro dopamine transporter density. BMC genetics. [online]..

[bib60] Wang G.-J. (2004). Decreased brain dopaminergic transporters in HIV-associated dementia patients. J. Neurol..

[bib61] Wang Y. (2020). Global prevalence and burden of HIV-associated neurocognitive disorder: a meta-analysis. Neurology.

[bib62] Womersley J.S., Seedat S., Hemmings S.M.J. (2017). Childhood maltreatment and HIV-associated neurocognitive disorders share similar pathophysiology: a potential sensitisation mechanism?. Metab. Brain Dis..

[bib63] Yavich L. (2007). Site-specific role of catechol-O-methyltransferase in dopamine overflow within prefrontal cortex and dorsal striatum. Off. J. Soc. Neurosci..

[bib64] Yen C.H. (2015). Reduced Dopamine Transporter Availability and Neurocognitive Deficits in Male Patients with Alcohol Dependence. PloS One. [online]..

[bib65] Yuan N.Y., Kaul M. (2019). Beneficial and adverse effects of cART affect neurocognitive function in HIV-1 infection: balancing viral suppression against neuronal stress and injury. J. Neuroimmune Pharmacol..

[bib66] Joint United Nations Programme on HIV/AIDS (UNAIDS). UNAIDS Data 2019; 2019. Available from: https://www.unaids.org/en/regionscountries/countries/southafrica. 12349391

